# Research Dissemination Strategies Used by Kenya Medical Research Institute Scientists

**DOI:** 10.24248/EAHRJ-D-18-00011

**Published:** 2019-07-30

**Authors:** James N Kariuki, Joysline Kaburi, Rosemary Musuva, Doris W Njomo, Doris Night, Carolyne Wandera, James Wodera, Pauline N Mwinzi

**Affiliations:** a Centre for Public Health Research, Kenya Medical Research Institute, Nairobi, Kenya; b Centre for Biotechnology Research and Development, Kenya Medical Research Institute, Nairobi, Kenya; c Centre for Global Health Research, Kenya Medical Research Institute, Kisumu, Kenya; d Eastern and Southern African Centre for International Parasite Control, Kenya Medical Research Institute, Nairobi, Kenya; e Marketing Department, Kenya Medical Research Institute, Nairobi, Kenya; f Corporate Affairs Department, Kenya Medical Research Institute, Nairobi, Kenya

## Abstract

**Background:**

Dissemination of research findings is acknowledged as an important component of any research process. Implementation of research findings into practice or policy is necessary for improving outcomes in the targeted community. Given the context and dynamic environment in which researchers operate, there is need to find out existing gaps in terms of disseminating research findings to key stakeholders. The objective of this study was to investigate the health research dissemination strategies used by Kenya Medical Research Institute (KEMRI) researchers.

**Methods:**

This was a mixed-method study employing concurrent sequence (use of both qualitative and quantitative) methods of data collection. The study was conducted in KEMRI's 10 centres spread in 3 geographical areas: Kisumu, Kilifi, and Nairobi counties. Potential respondents were identified through purposive sampling. Three inter-related data collection methods were employed in this study. These methods included key informant interviews with: (a) MoH officials from county government; (b) KEMRI researchers; and (c) key KEMRI departments, namely Corporate Affairs and the library. Additionally, secondary sources of information, such as scientific reports, KEMRI annual reports, and financial statements, were also reviewed.

**Results:**

Publication of papers in peer-reviewed journals was mentioned as the most common method of dissemination of research findings. Scientists published in 353 peer-reviewed journals (or publishing houses) between the years 2002 and 2015. Over 92.7% of these publications were in international peer-reviewed journals. Conferences and workshops were also mentioned. In the absence of a centralised electronic KEMRI publication database, the research team extracted and collated a publication lists from KEMRI annual reports and financial statements. This was limiting since it did not have an exhaustive list of all publications by KEMRI scientists. Only 3 respondents mentioned having written policy briefs or engaged the media as part of dissemination channels. The media representatives cited the use of social media (Facebook and Twitter) as another channel that KEMRI scientists could exploit. Challenges in dissemination included lack of knowledge on research translation leading to poor synthesis of research outputs as well as selective reporting by the media.

**Conclusion:**

Publications in peer-reviewed journals was the most preferred channel of communicating scientific outputs. Conferences and writing of policy briefs were the other sources of dissemination. We recommend that KEMRI dissemination channels should go well beyond simply making research available through the traditional vehicles of journal publications and scientific conference presentations but establish institutional mechanism which would facilitate extracting the main messages or key implications derived from research results and communicating them to stakeholders in attractive ways that would encourage them to factor the research implications into their work.

## INTRODUCTION

Dissemination is acknowledged as an important component of the research process. The dissemination and implementation of research findings into practice is necessary so as to apply research findings to improve outcomes in the broader community.^1^ Innovative models to facilitate more rapid uptake of research findings into practice are urgently needed.^2^ Previous studies indicate that a number of research findings which if implemented would have significantly improve health or behavioural outcomes, failed to be translated into meaningful public health interventions across multiple contexts.^3,4^ Barriers to dissemination and implementation may occur at multiple levels of research and health-care delivery namely at the researcher level, patient level, organisational level, or the market/policy level.^5^

Moving the field of scientific dissemination forward will require studies that identify mechanisms and approaches to package and convey the evidence-based information necessary to improve public health and clinical care services in ways relevant to local settings and that balance fidelity and adaptation.^3^ Nonetheless, the communication of research findings in a rural sub-Saharan African setting is less straightforward and presents significant challenges with respect to literacy, language, logistics, and confidentiality. In recent years, the Internet and television have revolutionised dissemination as well as introduced new sets of challenges.^6^ There is need to find out what the challenges are in disseminating research findings, from researchers, Ministry of Health (MoH) officials and the media, who are key stakeholders in this process.

Interventions developed in the context of efficacy and effectiveness trials are rarely transferable without adaptations to specific settings and additional tools and guidance to support uptake and implementation. Therefore, research is needed to examine the process of transferring interventions into local settings, which may be similar to but also somewhat different from the ones in which the concepts were developed and tested. The most prevalent strategy for dissemination has been to target scientists to increase their dissemination efforts. A combination of education, incentives, and admonishments are required to encourage health scientists who develop and test incentives to also find innovative ways to disseminate results. This approach has however been criticised as being misguided on the basis that asking scientists to be central players in dissemination when they lack the necessary training and usually operate in organisational environments that lack the infrastructure and reward structure to motivate and support systematic dissemination, is unrealistic.^6^

As a necessary prerequisite for unpacking how information which can lead to intervention or service changes, we need to understand how and why information on physical and behavioural health, preventive services, disease management, decision making, and other interventions may or may not reach various stakeholders, or why they might not be able to utilise it when it reaches them. We need to understand what underlies the creation, transmission, and reception of information on evidence-based pharmacological, behavioural, genomic, policy and systems interventions.^6^ Successful dissemination of health information (including evidence about underutilised interventions) may occur quite differently depending on whether the audience consists of consumers, caregivers, practitioners, policymakers, employers, administrators, or other multiple stakeholder groups.^7^ Unless health research findings are communicated effectively, there will be a little chance of those changes happening.^8^ The question arises as to how those findings should be disseminated to them in a suitable format when they become relevant. By practice, it is known that researchers at the institute publish their finds in various journals, but to the best of our knowledge, this has not been documented. The objective of this study was, therefore, to investigate and document dissemination strategies used by Kenya Medical Research Institute (KEMRI) scientists and their effectiveness to stakeholders.

## METHODS

### Study Area

This study was conducted in KEMRI's 10 centres located in Nairobi, Coast and Western Kenya. The institute is a state corporation established by an Act of Parliament as the national body responsible for carrying out research for human health in Kenya. The majority of these centres are located in Nairobi County, and they include the Centre for Biotechnology Research and Development (CBRD), Centre for Clinical Research (CCR), Centre for Microbiology Research (CMR), Centre for Public Health Research (CPHR), Centre for Traditional Medicines and Drug Research (CTDMR), Centre for Virus Research (CVR), Centre for Respiratory Diseases Research (CRDR), and Eastern Southern Africa Centre for International Parasitic Control (ESACIPAC). Other centres outside Nairobi include: Centre for Global Health Research (CGHR) in Kisumu County, Centre for Geographic Medicine Research – Coast (CGMR–C) in Kilifi County and Centre for Infectious and Parasitic Diseases Control Research (CIPDCR) in Busia County. MoH programme managers were targeted in the 4 counties where the KEMRI Centre are located. Journalists from media houses in Nairobi were purposefully selected for inclusion into the study.

### Study Design

This was a mixed methods study employing a concurrent sequential method of data collection. That is, it involved the collection of qualitative and quantitative data simultaneously. A semistructured questionnaire and interview guide were the 2 tools that were used to collecte quantitative and qualitative data, respectively.

### Study Population

The total number of research staff as at the time of conducting the survey were 250 scientists who have diverse qualifications and skills in matters of health. In addition, there are over 300 technologists and technicians who provide research support to the scientific community. All the research scientists were eligible for consideration to participate in the study.

### Sampling

The study established that at the time of undertaking the survey, a number of research officers were either out in the field collecting data or were officially on leave. Thus, all the researchers who were found at their respective workstations were interviewed. No sampling of respondents was necessary. Potential respondents of the in-depth interviews were identified through purposive sampling. Researchers were identified on the basis of i) Principal investigators with more than 1 study concluded, ii) Scientists from the same centre working on different disease profiles to give diversity on thematic areas of interest iii) Scientists who have been in KEMRI for more than 7 years to give depth on issues of dissemination iv) Scientists who provided informed verbal consent. Also included in the interview list, were respondents from KEMRI's Corporate Affairs Department and the Library. Additional interviews were also carried out with health journalists from established media houses, as well as MoH representatives/heads of policy development departments at the county government levels to assess their uptake of health research findings from KEMRI researchers.

### Data Collection Methods

The following 3 data collection methods were employed in this study. In-depth interviews with MoH officials from the county government as well as key KEMRI departments (Corporate Affairs and the library). Review of secondary sources of information such as scientific reports and KEMRI annual reports and financial statements. In-depth interviews with KEMRI researchers

#### In-depth Interviews with MoH Officials

An interview guide containing questions addressing the broad areas of the baseline study was formulated. These themes included 1) policy changes implemented in the last 10 years; 2) what influenced policy change; 3) interaction with KEMRI; (4) views on how interactive with KEMRI could be improved; 5) What research from KEMRI had benefited their work or any interventions they had implemented. Sociodemographic information of respondents was also collected. A total of 3 KIIs were carried out and were conducted in English.

#### Review of Secondary Sources of Information

In the absence of a centralised electronic KEMRI publication database, the research team extracted and collated a publication list from previous KEMRI annual and financial statements reports from the year 2002 to 2016. These annual and financial reports contained a list of publications by staff as part of the annexure section for each year under review and thus provided an objective and verifiable source document. However, it was found to be limiting since it did not have an exhaustive list of all publications by KEMRI scientists and in some instances had duplication of publications by the same authors. To examine the preferred journal and content of KEMRI publications, a content analysis was performed on papers published. A content analysis provided a means for objective, systematic, and quantitative consideration of published articles. It also allowed for an interpretation of the direction in which KEMRI scientists are taking in terms of priorities of publications. Two reviewers examined the list of publications and coded them into pre-determined themes. A third reviewer was contacted whenever there was a disagreement.

#### Interviews with KEMRI Researchers, KEMRI Corporate Department, and Library

Key informant interviews (KIIs) were carried out targetting specific departments. The checklist consisted of questions relating to dissemination practices, preferences, and future demand for KEMRI research outputs. This survey targeted to conduct at least 5 KIIs per group, with an option of conducting more until a point of response saturation was attained. The main focus in these guides included methods used for data sharing; challenges in dissemination of research findings; interaction with the media and MoH; how that interaction can be improved; factors that have influenced research use in policy making; factors that have hindered research use in policy making; health issues popularly published; interaction with KEMRI researchers; research packaging by KEMRI scientists.

### Data Management and Analysis

Qualitative data were transcribed verbatim. The team of qualitative researchers first familiarised themselves with the transcripts, after which independent coding was done and the codes collectively finalised for each tool. In case of a disagreement on a theme, a third party was called to break the deadlock. The conceptual model for considering diffusion, dissemination and implementation of innovations in health service delivery^9^ was used to tease out categorisation of the data collected.

### Ethical Approval

Before the commencement of the survey, scientific and ethical approval was sought and received from the national Scientific and Ethical Review Unit (SERU), based at KEMRI. In addition, approval was sought from the directors of each of the 10 centres that constitute KEMRI. During the interview process, informed consent was obtained from the study participants. Additional consent was sought when interviews were to be tape-recorded. Permission to publish this manuscript was also received from the KEMRI Director's Office.

### Data Limitation

At the time of conducting this survey, a number of scientists were out of their workstation on official duties. Repeated visits to the stations did not yield much in terms of interviewing more staff members. This was a limitation, especially when compiling the findings. In addition, KEMRI did not have a centralised electronic publication database which would facilitate data mining. The researchers were referred to online journals so as to compile an institutional list of publications. This resulted in duplication of effort. Furthermore, scattered database and profiles were located in different Centre and departments. The Monitoring and Evaluation (M&E) Department of KEMRI had a more organised but not exhaustive list of staff publications. The list of publications from this department formed the basis of secondary desktop review as it was conveniently located.

## RESULTS

### Quantitative Findings

A total of 37 KEMRI scientists were interviewed during the survey. Their sociodemographic profiles are shown in Table 1.

**TABLE 1.** Sociodemographic Characteristics (N=37)

Description	n (%)	95% CI
**Sex**		
Female	11 (29.8)	17.5%–45.8%
Male	13 (35.1)	21.8%–51.2%
Did not disclose^a^	13 (35.1)	21.8%–52.1%
**Age in years**		
30-34	2 (5.4)	1.5%–17.7%
35-39	4 (10.8)	4.3%–24.7%
40-44	4 (10.8)	4.3%–24.7%
45-49	4 (10.8)	4.3%–24.7%
Above 50	10 (27.0)	15.4%–42.9%
Did not disclose^a^	13 (35.1)	21.8%–51.3%
**Educational level**		
Secondary	1 (2.7)	0.4%–13.8%
College	2 (5.4)	1.5%–17.7%
Bachelor's	2 (5.4)	1.5%–17.7%
Master's degree	7 (18.9)	9.5%–34.2%
PhD	18 (48.7)	33.5%–64.1%
Did not disclose^a^	7 (18.9)	9.5%–34.2%

a Represents the number of respondents that did not give responses with regard to sex, age, and education level

Abbreviation: CI, confidence interval

#### Publications in Peer-Reviewed Journals

A total of 1,639 publications were published by KEMRI researchers between the period 2002 to 2016. During the period under review, KEMRI's scientists published in 353 peer-reviewed journals of which 92.7% were published in international journals. The East African Medical Journal was the only journal from a developing country listed among the top 10 preferred journals, accounting for 7.3% of KEMRI publications. Among the top 10 journals preferred by KEMRI researchers, the *PLoS* series of journals accounted for 18.7%, *Malaria Journal* (13.7%), while the *American Journal of Tropical Medicine and Hygiene* accounted for 12.8%, as shown in Table 2.

**TABLE 2.** The Top 10 Peer-Reviewed Journals in Terms of KEMRI Publications Between 2002 and 2015

Peer-Reviewed Journals	Number Published by KEMRI Scientists	Percentage	Impact Factor Information	
			Impact Factor	Notes/Source of Information
**PLoS series of Journals**	138	18.7 %	-	PLoS does not consider Impact Factor to be a reliable or useful metric to assess the performance of individual articles.
**Malaria Journal**	101	13.7 %	3.079	malariajournal.biomedcentral.com/about
**American Journal of Tropical Medicine & Hygiene**	94	12.8 %	2.740	http://www.ajtmh.org/journal-facts
**BMC series of Journals**	65	8.8 %	-	The BMC series is a collection of high-quality, peer-reviewed journals covering all areas of biology and medicine, focusing on the needs of the research communities which they serve.
**Tropical Medicine & International Health**	71	9.6 %	2.519	http://onlinelibrary.wiley.com/journal/10.1111/(ISSN)1365-3156
**Journal of Infectious Diseases & Immunology**	65	8.8 %	1.69	www.esciencecentral.org/journals/infectious-diseases-and-therapy.php
**Lancet series of Journals**	65	8.8 %	21.372	www.journals.elsevier.com/the-lancet-infectious-diseases/
**East Africa Medical Journal**	54	7.3 %	0.11	www.researchgate.net/journal/0012-835X_East_African_medical_journal
**AIDS Journal**	42	5.7 %	5.554	en.wikipedia.org/wiki/AIDS_(journal)
**Transactions of Royal Society of Tropical Medicine & Hygiene**	42	5.7 %	1.909	academic.oup.com/trstmh/
**Total**	**737**	**100%**		

All the respondents (n=37) reported they also attend and present the findings of their research outputs at international conferences. The choice of which conference to attend and funding depends on researchers preferences and the availability of additional funds. The most commonly mentioned conference include American Society of Tropical Medicine and Hygiene (ASTMH) Annual Conferences (48.7%) as well as the annual Pan Africa Mosquito Control Association (PAMCA) conference (27.0%). Table 3 profiles the most commonly attended conferences as reported by the respondents.

**TABLE 3.** Most Commonly Mentioned Conferences and Seminars Attended by KEMRI Staff to Disseminate Research Findings (N=37)

Name of Conference	Meeting Location (Local/Regional/International)	Host/Convener	n (%)^a^
**American Society of Tropical Medicine and Hygiene (ASTMH) Annual Conference**	International	American Society of Tropical Medicine and Hygiene (ASTMH)	18 (48.7%)
**Drugs for Neglected Diseases Initiative (DnDi) Annual Conference**	International	World Health Organization/DnDi Programme	12 (32.4%)
**Pan Africa Mosquito Control Association (PAMCA) Annual Conference**	International	PAMCA	10 (27.0%)
**The Union World Conference on Lung Health**	International	The International Union Against Tuberculosis and Lung Disease	8 (21.6%)
**MoH-related conferences/workshops/seminars**	Local	Various departments of the Ministry of Health, Kenya	16 (43.2%)
**KEMRI Annual and Scientific Conference (KASH)**	Local (hosted by KEMRI)	KEMRI	15 (40.5%)
**East African Health and Scientific Conference and Medical Exhibition**	Regional	East African Health Research Commission (EAHRC)	5 (13.5%)
**African Society for Laboratory Medicine (ASLM) Annual Conference**	Regional	(ASLM)	3 (8.1%)

a Some participants provided multiple responses.

### Qualitative Findings

Publication of papers in peer-reviewed journals was the frequently mentioned method of dissemination of KEMRI research findings. Other dissemination channels included presentations at conferences, seminars, workshops and generation of reports to KEMRI and research clients. Only a few participants mentioned having written policy briefs or engaged the media.

**Male researcher, Kilifi Centre: “*Scientists don't have training in writing media and policy briefs. That could be 1 reason why we don't use those methods… I think there are many levels of approval before one can use the media. That is discouraging, so mainly we will just publish in journals*”.**

Respondents reported that they were not motivated to publish. The numbers of publications had no influence on job promotions or assignment of responsibilities.

**Female researcher, CPHR: “…*honestly can't say that as a KEMRI scientist I am motivated to publish. We do it because it's part of the job. The promotions are not even based on that. You will see someone with 1 publication getting promoted and another with 10 getting stuck... We get more recognition outside than right here*”.**

Respondents involved in the IDIs expressed their frustrations with journals citing long turnaround periods, which sometimes render data obsolete. Other issues of concern included a lack of knowledge about research translation leading to poor synthesis, limited funding to attend conferences, and selective reporting by media.

**Male researcher, CMR: “…*sometimes I think we people in science talk to ourselves and I think it is important for us to learn to simplify our language and our findings so that you know it is usable to the other people*”.**

**Male researcher, C-GHRC: “*I think there is a lot of bias. Coverage will be given to Zika virus, Ebola or when there is an outbreak of a disease… So you will find others equally detrimental to health are left out*”.**

The majority of researchers pointed out that there is a disconnect between the KEMRI departments responsible for research dissemination and the centres, which further aggravates the lack of research being taken up as policy or practice.

**Female researcher, CRDR: “*There is a department… which is supposed to link us to the media or people out there. They are the ones to take up the issue. Now that department has not been doing that. I have not heard*”.**

**Male researcher, CBRD: “*Now if someone is working in that department and they don't come around they don't find the interesting finding that is 1 reason why it has not worked… there is a disconnect*”.**

**Male researcher, CTDMR: “*… there is a particular department… like now in marketing. You should take [up] the challenge because during eeh… events that is where you should engage the KEMRI scientist to come and maybe speak or talk about what they are doing. KEMRI is a research institution, so why are you not engaging the scientist in every one of those activities?*”**

#### Policy and Practice Changes Impacted by Research Done at KEMRI

In-depth interviews with KEMRI scientists revealed that most of their research had influenced changes in policy and practice in the country. It was interesting to note that this view was not necessarily acknowledged by the MoH officials. The MoH did not attribute any changes in policy and practice with research done at KEMRI. This could partly be attributed to the frequent reshuffling of officers in the various ministries as well as limited access to published material, as mentioned by the respondents.

**Male researcher, CCR: “*I can't boast about it as my work, but together with others it has contributed like change of policy from chloroquine to SP, from SP to ACTs and right now we are working on the issue of correcting ACTs into schistosomiasis. It's still on an early stage, but I believe that there are discussions on very high levels… even the transfusion guidelines in Kenya. The studies that we did in Siaya yeah have contributed into those guidelines because initially, it was like if you have haemoglobin of 5, but our studies showed transfuse the patient and not the lab result. Yeah (Laughs)*”**

**Male researcher, CVR: “*For example, look at the HIV testing among infants that started as a research thing around here initially around 2006 all the way to 2008. Do you know that programme was taken up by the ministry, and now it is a national programme that's how the infants are being tested for HIV all over the country? That's a clear area that showed that research showed that this can work because that is molecular testing*”.**

#### Barriers to KEMRI Research use by Decision Makers

Majority of the MoH officials and media journalists mentioned poor synthesis of research as a major factor contributing to research not being taken up as policy or practice. The scientific language limits the audience to fellow researchers who may not necessarily have a say in policy direction, thus the gap.

**Female Journalist, 31 years:” *well eeh.. scientist you usually communicate in a very technical language, and journalist eeh communicate in a very simple easy to understand language everybody can understand so aaaah I know the scientist eeh… communicate in that kind of language because of the nature of their work. It will be good for them if they communicate in a language that eeh it is eeh friendly to the journalist and… for to the public*”.**

**Male researcher, EASCIPAC: “… *I think it is a problem everywhere. Scientists conduct their research all the time, everywhere in the whole world, but… translating this item into policy findings… there is a disconnect between the researchers and the policy makers. Sometimes even the policy makers do not understand your language so I know in certain institutions they form partnerships with private companies to uptake the data coming from their scientists and convert them into a product that is sellable so that way we are not saying we gave our data to the Ministry of Health… and they did not act on it so KEMRI in itself through this private companies can actually make a product out of it*…”**

**Female respondent, 33 years, MoH: “*You know, we don't have access to Internet here… people don't know where to find those journals… Even the reports that people bring here are collecting dust. But if you come to the office, call the officers concerned and share your results, then I think that's the best way to proceed. People can ask questions, and everyone is satisfied and understands what it is about*”.**

Other impediments mentioned included the choice of dissemination method, financial implications involved in implementing policy changes, donor-driven research that does not address local needs, priorities of media house and policy makers, delays in ethical clearance from KEMRI and ‘media phobia’ from scientists.

**Female Journalist, 31 years: “*I don't know whether the scientists have been sensitised about how to deal with the journalist or they do not know how the journalist profession works… so they are quite hesitant when it comes to providing this research information that the scientist has undertaken. So much valuable information is not out there because scientists are afraid to talk to journalists. We need to work together*”.**

**Female researcher, CCR: “…*for donor-driven research, it mostly starts as a collaboration, but later, they want to bully and boss you out, even overtake you as the local researcher and run the show. Now, in the end, your objective becomes a small component of the study. So when you want to sell the idea, no one buys it… Because what does it address anyway?*”**

**Male researcher, CBRD: “*We do not have experience or training in writing policy briefs or media briefs so in the end, who are we targeting? We will publish in peer-reviewed journals, but not everybody has access to that. Not everyone is going online to look. So there is a gap; there are important people not accessing this data. How will it even inform policy then?*”**

#### Suggested Way Forward by Researchers

Researchers mentioned the need to have systems put in place in KEMRI that ensures dissemination of research results. Another key factor mentioned was that researchers need more training on re-packaging of findings to improve chances of research products and outcomes being taken up as policy or practice. Other factors mentioned included functional links between the KEMRI researchers and the corporate department; advocacy for KEMRI research findings to partners and stakeholders; having in place a digital repository in the library; and use of social media.

**Male respondent, 45 years, MoH: “*There was a time representatives from KEMRI used to attend our meetings, and it worked well because we were informed of what the scientists are doing. That was a while back. KEMRI now has no visibility here*”.**

**Male researcher, CPHR: “*You see research and policy are somehow detached, especially where institutions don't work like together they are working as separate entities. So for us, I think one of the things is to become proactive in all the areas like doing a lot of lobbying*…”**

**Female Librarian: “…*we should be able to reflect on what KEMRI does and what KEMRI has been doing for the past, and it would just be nice if someone can access from wherever. We need a digital institutional repository which will work hand in hand with the digital library. I believe, if implemented, it will create a good working information library system that will now uplift our digital level on the electronic part… once we start working with departments, we will be able to get information from centres and the researchers. The repository will bring this together*…”**

## DISCUSSION

This study provides insights into strategies used by KEMRI researchers and barriers that hinder the dissemination of research findings. The insights are summarised as follows.

### Dissemination Channels

This survey established that KEMRI scientists' most preferred avenue of dissemination is through publication in peer-reviewed journals. For researchers, the assessment of productivity and contribution to science is highly pegged by quantifiable means such as publications. Given that the success of a scientific paper partly depends on its outcome, researchers tend to publish their findings in high impact peer-reviewed journals^10, 11^ as well as in open access options^12^ that provide the likelihood of it being cited by other authors. By extension, publications that appeared in high-end peer-reviewed journals were associated with knowledge prowess on a particular subject or discipline. Apart from contributing to the knowledge base, publications also inform tenure and future funding directions.^13^

### Best Practices in Dissemination of Research Findings

Only 3 (8.1%) scientists reported that they had exposure to media engagement (television and radio shows). From the findings, the publication of research findings in local print and electronic media was limited. Use of social media was cited as another channel that is becoming popular with KEMRI scientists. This survey did not establish the impact of the use of social media on the dissemination or advertisement of research findings.

### Uptake of Health Research Findings

This study established that there was a disconnect between researchers' work contributing to national policy formulation and inputs into decision making processes. Scientists pointed out circumstances in which their outputs were used to inform policy and practice. However, the potential consumers of KEMRI's research findings, namely the policy makers and journalists reported that they did not share this view. Synthesis of research into policy/practice by government bodies, organisations and other stakeholders is gravely undermined by the different levels of research awareness and experiences within these teams.^14^

Barriers to research dissemination and implementation may occur at multiple levels, namely individual researcher level, organisational, and at market/policy level.^15^ These barriers are discussed in the subsequent paragraphs.

### At Individual Level

The instructions to authors usually guide the scientific language to be used and how the information is packaged.^16^ Many researchers have limited exposer to media. Only a handful of scientists have had previous training in writing and handling media. A strategy is required to overcome ‘*media phobia*’ by scientists. Potential users of research outputs face challenges of synthesising research articles arising from various KEMRI publications. This is consistent with studies conducted elsewhere.^15, 17^ This problem is partially aggravated by the high impact journals which have structured guidelines that emphasis on form-over-substance.

### Organisational Level

Prior to a change in policy directive, all publications and related outputs had to seek ethical approval from the Office of Director KEMRI. This resulted in publication delays and a backlog of manuscripts, as researchers sort additional authority-to-publish from the institute. By the time of undertaking this survey, there was a policy directive that manuscripts should be cleared for publication by the centre scientific committees. This was aimed at reducing pile-ups of manuscripts and the time lags that it takes to publish them. One of the participants mentioned that a number of studies carried out in KEMRI are funded by external donors, hence by extension, they partially determine the type of research to be conducted as well as where the findings will be published. Empirical studies have augmented that local utilisation of research outputs will occur once research can address local needs.^18, 19^ This can only be realised if the national and county governments prioritise their research needs and source for funding for the same.

### Market and Policy Level

The current survey established that priority changes by policy makers and preferences to certain health stories also contributed to the “slow” uptake of KEMRI researchers. Usually, these changes and preferences are not communicated to researchers. This gap probably explained why many KEMRI publications are not used to inform policy and practice.

## RECOMMENDATIONS

From this study, the following are the recommendations:-

1. Establish knowledge management and knowledge translation mechanisms at the institute to facilitate the collation, synthesis, packaging, and communication of research findings to decision makers and members of the public.
2. Encourage extensive use of social, online, and print media. This will offer a convenient way of accessing evidence anywhere at any given anytime. These platforms will also offer the chance of a back-and-forth engagement and not just passive dissemination.
3. Continue building on existing dissemination structures and processes which can help the uptake of research outputs. These include the annual KEMRI Annual Scientific Health (KASH) conferences and use of in-house bulletins such as the Bulletin and the Researcher. These will act as aids towards influencing decision making processes, especially when policy makers and implementers require evidence within the shortest period possible.

## CONCLUSION

Dissemination strategies at KEMRI should go well beyond making research available through the traditional vehicles of journal publications and scientific conference presentations. This survey established that there are a number of publications generated for local context were of high quality (methodology). Thus, we postulate that it is not the absence of information, but lack of an institutional mechanism which would facilitate extracting the main messages or key implications derived from research results. The re-packaged or synthesised research publications would possibly be communicated effectively to targeted groups of decision makers and other stakeholders using innovative ways as this would encourage them to factor the research outputs into policy formulation as well as guide practice.
